# Prediction of sequences of organ dysfunction in critical patients studied with Bayesian analysis

**DOI:** 10.1186/cc12417

**Published:** 2013-03-19

**Authors:** M Sandri, P Berchialla, D Gregori, L Gottin, V Sweiger, E Polati, RA De Blasi

**Affiliations:** 1 University of Verona, Italy; 2 University of Torino, Italy; 3 University of Padova, Italy; 4 University of Rome 'La Sapienza', Rome, Italy

## Introduction

The multiorgan dysfunction syndrome (MODS) is a dynamic process involving simultaneously or consecutively two or more organ systems [1]. The organ dysfunction's degree can be assessed by three severity scores (SOFA [2], MODS [3], LODS [4]), but they have some limitations: they do not allow the evaluation of the clinical course of a patient, they are not reliable in populations different from the reference one, and they do not support clinicians' decisions. Because MODS implies a systemic inflammatory reaction leading to microcirculatory dysfunction, our hypothesis was that organ failures follow a predictable sequence of appearance. Our aims were to verify the presence of more likely organ failure sequences and to assess an online method to predict the evolution of MODS in a patient. The high mortality and morbidity rate of MODS in ICUs can in fact be reduced only by a prompt and well-timed treatment [5].

## Methods

We selected 73 patients consecutively admitted to the ICU of Sant'Andrea Hospital from January to June 2012. The inclusion criteria were at least two organ systems with SOFA ≥2, ICU length of stay >48 hours. For each patient we calculated the SOFA since the beginning of the inclusion criteria and daily for 8 days. For the statistical analysis we used Dynamic Bayesian Networks (DBNs) [6]. DBNs were applied to model SOFA changes in order to identify the most probable sequences of organs failures in a patient who experienced a first known failure.

## Results

We created a DBN for the analysis of MODS studying the relations between organ failures at different times. The DBN was made so that each organ failure is dependent on the previous one. We also considered a corrective factor to take account that not all patients completed the observation. Using software (GeNie) we obtained the probabilities of the organ failure sequences.

## Conclusion

The use of DBNs, although with our limited set of data, allowed us to identify the most likely organ dysfunction sequences associated with a first known one. Capability to predict these sequences in a patient makes DBNs a promising prognostic tool for physicians in order to treat patients in a timely manner, or to test a treatment efficacy.
